# Predictors of homework engagement in internet-delivered Parent-Child Interaction Therapy for children with developmental delay: what about acculturation and enculturation?

**DOI:** 10.3389/frcha.2025.1500742

**Published:** 2025-03-12

**Authors:** Anastassia Cafatti Mac-Niven, Jonathan S. Comer, Daniel M. Bagner

**Affiliations:** ^1^ Department of Psychology, Florida International University, Miami, FL, United States; ^2^ Center for Children and Families, Florida International University, Miami, FL, United States

**Keywords:** homework engagement, acculturation, enculturation, Parent-Child Interaction Therapy, developmental delays, minoritized youth, culturally tailored engagement, telehealth

## Abstract

**Introduction:**

Families from racial/ethnic minoritized backgrounds and families of children with developmental delay (DD) often face more obstacles to engaging in psychosocial interventions compared to White families and families of typically developing children. Yet, research on engagement in behavioral parenting interventions has predominantly focused on typically developing children and White families from majority cultural groups. The present study offers the first examination of acculturation and enculturation as predictors of homework engagement among caregivers of children with DD from underrepresented racial/ethnic backgrounds participating in a telehealth behavioral parenting intervention.

**Methods:**

Data were collected from 65 caregiver-child dyads participating in the Advancing Child Competencies by Extending Supported Services (ACCESS) Study evaluating Internet-delivered Parent-Child Interaction Therapy (iPCIT) for children with DD. Homework engagement was measured as the proportion of days caregivers practiced “special time” with their child. Acculturation and enculturation were assessed using the Abbreviated Multidimensional Acculturation Scale (AMAS). Linear regression analyses evaluated associations between these two cultural factors and subsequent homework engagement, controlling for caregiver nativity, language of preference, income-to-needs ratio (INR), and caregiver work status.

**Results:**

While higher levels of acculturation (*B* = .110, *p* = .054) did not significantly predict homework engagement, enculturation (*B* = .140, *p* = .007) significantly predicted greater homework engagement throughout treatment with small and small-to-medium effect sizes (Cohen's *f*² = 0.029 and 0.104, respectively).

**Discussion:**

These findings underscore the nuanced role of acculturation and enculturation in predicting homework engagement in telehealth behavioral interventions for children with DD. Although acculturation did not facilitate homework engagement, caregivers who retained a stronger connection to their cultural heritage demonstrated higher homework engagement within the context of iPCIT. The study highlights the need for incorporating cultural considerations into treatment planning and flexibility in adapting treatment protocols to optimize family engagement and improve outcomes in this population.

**Clinical Trial Registration:**

ClinicalTrials.gov, identifier (NCT03260816).

## 1 Introduction

Externalizing behavior problems are common in young children with developmental delay (DD) (1, 2) and lead to long-term challenges in academics, social skills, interpersonal relationships, and self-care (3, 4). Behavioral parenting interventions (BPTs) are evidence-based treatments for early externalizing behavior problems (5, 6) that have shown to be effective for children with DD (4). Despite the efficacy of BPTs such as Parent-Child Interaction therapy in reducing externalizing behavior problems in children with DD (7–9), research shows families from racial/ethnic minoritized backgrounds experience more challenges engaging in psychosocial interventions than their White counterparts (10). Such treatment engagement challenges may be related to the increased stress observed in ethnic minoritized families of youth with DD, particularly when caregivers have reported greater challenges acculturating to the dominant culture (11–13). However, studies examining engagement in behavioral parenting interventions have been largely limited to typically developing youth and children from predominantly White families. The present study offers the first examination of acculturation and enculturation as predictors of engagement with behavioral parenting intervention services among caregivers of children with DD from predominantly underrepresented racially/ethnically diverse backgrounds.

Treatment effects of these BPTs have been shown to vary based on level of caregiver engagement in treatment, such as homework adherence (14–16). While BPTs, such as Incredible Years Parent Training (8) and Stepping Stones Triple *P* Positive Parenting Program (17), have also shown reductions in child externalizing behavior problems and caregiver stress (8, 17), PCIT's unique session structure and emphasis on building competence in specific parenting skills, such as the use of PRIDE skills and effective commands, sets it apart from other BPTs. Notably, PCIT's use of structured homework aims to reinforce these skills at home and support caregivers in applying them consistently beyond the clinical setting (7, 16). Given that caregiver engagement, including homework practice, is important for treatment success in PCIT, examining factors that may hinder or facilitate engagement, especially cultural identity variables such as acculturation and enculturation, is essential.

Homework in PCIT allows caregivers to practice parenting skills taught during session in real-life contexts, serving as an essential component of treatment adherence and facilitation of out-of-session skill generalization. Although previous studies included homework as part of a broader construct of caregiver engagement (16, 18), it is important to examine homework separately for several reasons. First, treatment is thought to be most effective when paired with consistent at-home practice, enabling families to generalize skills into daily life. The caregiver's ability to incorporate learned skills into the family's routine is considered important for treatment success. Second, observing positive behavioral changes in the child during homework practice can serve as a reinforcer for caregivers to continue to use the new skills. Third, homework practice provides an opportunity for caregivers to share with their therapist challenges that arise when practicing the skills and helps the therapist personalize treatment to address the unique needs of each family. Despite PCIT's focus on homework, research examining homework engagement specifically among families of children with DD remains limited (9).

Past research has provided support for the use of homework. For example, parents who completed more homework following a community-based PCIT intervention, demonstrated greater levels of treatment satisfaction, and a trend towards significance in predicting post-treatment child conduct problems (19, 20). However, studies examining homework have mostly excluded children with DD (20) despite the finding that children with DD initiate play interactions with their caregivers less often and respond to play with their caregiver differently than children without DD (21–23). To our knowledge, only one study examined the effect of homework in children with DD and found increased levels of homework engagement following clinic-based PCIT predicted lower levels of caregiver reported child externalizing behavior problems and parenting stress, as well as higher levels of observed positive parenting skills (9).

Despite the documented importance of homework practice, studies report average rates of homework engagement at just 50% or less (24–26). Caregivers have reported several barriers to homework, such as forgetting, not having support at home, lack of time outside of session, life stressors, and not agreeing with homework rationale (27, 28). These barriers are especially common among caregivers from low socioeconomic backgrounds and who identify as being from racial/ethnic minoritized groups (29–31). Increased barriers in these populations may be related to important cultural differences and less favorable perceptions of mental health treatments (29, 32). Although studies have shown promise for adapting PCIT to increase engagement among families from underrepresented racial/ethnic minoritized backgrounds (29, 33–35), none of these studies examined cultural identity predictors of homework engagement that might be especially relevant for these families, such as acculturation and enculturation.

*Acculturation* refers to the psychological process by which a member of a minoritized group adopts, acquires, or adapts to a majority or dominant culture (36). The process of acculturation is often complicated by systemic factors that minoritized families commonly face, including low socioeconomic status (SES), limited access to healthcare, intergenerational trauma, language barriers, mistreatment, and distrust in mental health services, as well as under recognition of mental health problems (37). These stressors create additional challenges for caregivers, that can affect families' active involvement and engagement in mental health treatment (38, 39) and other mental health services. For example, caregivers from low SES backgrounds may struggle with financial and logistical barriers that hinder their ability to participate in treatment. Similarly, language barriers may limit effective communication with providers, leading to misunderstandings and therefore decreased engagement. Previous research shows that lower levels of acculturation predicted increased levels of caregiver stress, particularly among caregivers of children with DD and from racial/ethnic minoritized backgrounds (12, 40, 41). There are additive stresses of adapting to a dominant culture and parenting a child with special needs and behavioral concerns (13). This heightened stress may further affect caregivers' ability to navigate the complexities of treatment, ultimately leading to lower homework engagement and less favorable outcomes. In fact, caregivers in samples with socially complex needs report a host of barriers to homework completion, leading to less frequent out-of-session practice despite the well documented benefits of completing homework (9, 27). Thus, we expected families reporting less psychological connection, identity, and facility with dominant U.S. culture (i.e., lower levels of acculturation) would report lower homework engagement during treatment.

Despite the challenges that arise from navigating cultural adaptation, it is important to highlight that the acculturative process is multifaceted, encompassing not only significant stressors but also opportunities for growth and resilience. While systemic barriers and acculturative stress can hinder engagement in treatment, research has also shown that acculturation can foster protective and beneficial outcomes, such as enhanced resilience among minoritized families, increased access to social capital, and expanded social networks (42, 43). Past research has also shown support for the immigrant health paradox, which shows less acculturated individuals have demonstrated protective health outcomes compared to their more accultured peers, potentially due to protective factors from their culture of origin (44). However, research has also shown that this paradox protection appears to be inconsistent across races, ethnicities, age groups, or genders (45). The effects of acculturation on physical and mental health studies to date continue to reveal discrepancies and contradictory results across different racial/ethnic populations (46, 47).

This dual nature of acculturation underscores the importance of considering both its challenges and potential benefits. The acculturative process can bring significant advantages, particularly when families develop bicultural competence, also known as integration. Adopting aspects of the dominant culture while maintaining ties to one's culture of origin seems to yield the most favorable outcomes, including enhanced engagement in mental health treatment (48–50). This study examines how both the challenges of acculturative stress and the strengths of cultural identity interact to influence caregiver homework engagement in PCIT. It is the combination of acculturation and enculturation that appears to offer the most protective factors.

*Enculturation*, a related but distinct construct (51), refers to the maintenance of identification or norms and customs from one's familial culture of origin (52), including traditions, language(s), beliefs, and values (53). Prior studies have shown that high levels of enculturation can be protective against mental health problems (12, 54, 55). For example, maintaining cultural values related to family and parenting, such as *familismo* (i.e., family cohesion and prioritizing the family's needs over individual needs) in Latinx/Latine families (56, 57), may be important to emphasize in treatment as they allow caregivers to rely on others for support while they engage in caregiving practices. Continuing to have these support networks may also assist caregivers in managing the challenges associated with acculturation. Thus, capitalizing on culturally oriented protective factors within treatment could be particularly important for minoritized families and marginalized populations. For these reasons, we expected families maintaining psychological affinity with their culture of origin (i.e., those reporting higher levels of enculturation) would report higher homework engagement during treatment.

Traditionally, acculturation was conceptualized as a unidimensional process, where maintaining one's heritage culture and adopting the values of a receiving or dominant culture were opposing poles on a single continuum (58). This outdated understanding implied that, for immigrant populations, acquiring values, practices, and beliefs of a host society inherently required the discarding of one's own culture of origin (48). Today, research suggests that acculturation is better understood as a bilinear, multidimensional, and context-dependent process of cultural socialization (55, 59). Rather than being mutually exclusive, acculturation and enculturation are now recognized as complex and dynamic processes that can occur simultaneously and interact in non-linear ways (12). In fact, Berry's model (60) demonstrates that these two dimensions intersect to create four different acculturation categories (i.e., high in acculturation and enculturation, low in both, or high in one and low in the other). Assimilation involves a full adoption of the dominant culture, with little to no retention of their original culture. Integration refers to the combination of elements of both the original and dominant culture. Separation involves retaining the original culture, while rejecting the dominant culture. Lastly, marginalization highlights the loss or rejection of both the original and dominant cultures. Importantly, integration, also known as biculturalism, where individuals adopt aspects of the receiving culture while maintaining their heritage culture, has been associated with the most favorable psychosocial outcomes, especially among young immigrants (48, 60). In this study, we aim to highlight the bidimensional nature of these processes, acknowledging that families can experience both high acculturation and enculturation synchronously.

Acculturation and enculturation could influence treatment engagement in iPCIT for several reasons. When it comes to acculturation, families with lower levels of acculturation may experience cultural dissonance between the principles emphasized in iPCIT vs. their own values. For example, strategies such as verbal praise and/or ignoring may conflict with culturally rooted norms that prioritize different forms of discipline or reinforcement such as the increased use of commands in Spanish-speaking Latinx/Latine caregivers (61–63) or the tendency to utilize harsher forms of verbal and physical discipline, for example within the African American and Latinx/Latine families (64). Additionally, the level of trust in mental health professionals, shaped by cultural perceptions of healthcare systems (65–67), may influence families' willingness to engage in the therapeutic process, including completing homework assignments.

High levels of enculturation, on the other hand, may enhance treatment engagement by serving as a protective factor. As previously mentioned, cultural values such as familismo in Latinx/Latine families, may motivate caregivers to prioritize their child's needs and adhere to treatment requirements, including homework, to benefit the family as a whole and to fulfill their role as responsible parents (56, 68). For Black/African American families, the value of resilience and communalism (e.g., strong sense of shared responsibility and collective well-being), may also motivate caregivers to adhere to treatment requirements, such as completing regular homework practice by prioritizing actions that ensure family stability and empowerment (69–71).

It is also important to examine how acculturation and enculturation may affect homework engagement in the context of a telehealth treatment. Most studies examining homework engagement in the context of BPTs have focused on clinic-based care. Increasingly, telehealth methods are being leveraged to expand the reach of behavioral parenting interventions to underserved populations [e.g., (72–75)]. Acculturation and enculturation may impact an individual's willingness to engage in telehealth services in multifaceted ways depending on their familiarity and comfort with technology. For instance, caregivers with lower levels of acculturation may face challenges such as limited technology literacy or distrust of digital platforms (76). Others may feel uneasy with the idea of exposing their living conditions on camera or having them or their child being recorded. Similarly, enculturation may play a role in shaping perceptions of telehealth. For example, families who adhere to more traditional cultural norms may prefer in-person interactions that align more closely with culturally rooted expectations for support or caregiving. On the other hand, the physical distance imposed by telehealth may reduce the stigma of receiving psychological services, which is a common barrier experienced by racial/ethnic minoritized families, and thus facilitating self-disclosure and acceptability of support (65–67). Understanding these dynamics among families from minoritized backgrounds is vital when examining new treatment modalities, as barriers to technology acceptance could lead to reduced treatment adherence, and consequently lower homework engagement, undermining the effectiveness of interventions designed to support children with DD.

Although one of the explicit goals of the telehealth format is to improve treatment engagement, to our knowledge, only one study to date has examined homework engagement in the context of telehealth parenting intervention services. Specifically, Sanchez, Javadi, & Comer (76) compared family engagement across clinic-based PCIT and Internet-delivered PCIT (iPCIT) (65) and found the telehealth format improved treatment attendance (particularly for families holding minoritized identities), but not homework engagement. However, this study only included typically developing children, the majority of the sample was made up of non-Latinx/Latine White families, and analyses did not consider cultural identity predictors, such as acculturation and enculturation. Much remains to be learned about homework engagement and associated cultural factors in the context of telehealth-based parenting treatments for children with DD.

To address a number of key gaps in the literature, the present study examined acculturation and enculturation as predictors of homework engagement in caregivers of children with DD from primarily underrepresented racial/ethnic minoritized backgrounds participating in iPCIT. Data were drawn from a randomized controlled trial (RCT) comparing iPCIT to referrals as usual (RAU) for the treatment of behavioral problems in children with DD (66). Given the challenges and stresses of having to navigate mental health services and treatment participation in the context of a dominant culture, we expected that caregivers reporting higher levels of acculturation would complete higher rates of homework across treatment. In addition, given the many observed protective functions of maintaining psychological affinity with one's culture of origin (12, 55, 67, 77, 78) we also predicted caregivers reporting higher rates of enculturation would complete higher rates of homework across treatment.

## 2 Materials and methods

### 2.1 Participants

Participating families were recruited as part of a randomized controlled trial (RCT)—i.e., the Advancing Child Competencies by Extending Supported Services (ACCESS) Study (66). Recruitment for this RCT occurred between March 17, 2016, and August 28, 2019 at three Part C Early Intervention (EI) sites in South Florida that provide services to children aged 0 to 3 years with DD. Specifically, recruitment for this clinical trial occurred during the child's EI exit evaluation within 3 months of the child's third birthday and when they aged out of eligibility for EI services. During their EI exit evaluation, children were assessed by their corresponding EI site utilizing the Battelle Developmental Inventory (BDI), Second Edition (79), to evaluate early childhood developmental milestones and help identify children at risk for DD. Inclusion criteria for the youth with DD and their caregivers were: (1) Child Behavior Checklist (CBCL) externalizing problems T score ≥ 60; and (2) primary caregiver spoke English and/or Spanish. Exclusion criteria were: (1) child receiving psychiatric medication for behavior problems; (2) child/caregiver deafness or blindness; (3) severe child social communication deficits (i.e., caregiver report on Social Responsiveness Scale, second edition, T score >75), although children with moderate social communication deficits (scores between 66 and 75), including those with autism spectrum disorder, were eligible; and (4) primary caregiver received a standard score <4 on the vocabulary subtest of the Wechsler Abbreviated Scale of Intelligence, second edition (for English speakers) or Escala de Inteligencia Wechsler Para Adultos, third edition (for Spanish speakers).

Although 150 caregiver-child dyads participated in the overall RCT only those randomly assigned to receive iPCIT (in English or Spanish) were included in the current study (i.e., *n* = 75 families), as families in the control condition were not assigned homework. Of those 75 families, one did not identify as being from a racial/ethnic minoritized background or from a culture of origin outside the United States (and thus acculturation and enculturation would not be relevant for this family), five families dropped out of treatment before completing any sessions, and four dropped out after the first session and before homework was assigned. Thus, a total of 65 families were included in the current study. As presented in Table 2, the mean developmental functioning scores for the children across the sample as measured by the BDI, was 75.58, which falls in the low average range and is considered “at risk” for DD (66, 79). Additionally, the mean child CBCL externalizing T score was 67.43, indicating that, on average, children in this study exhibited behavioral problems within the clinical range (66). Most of the children (73.8%) were boys, and most of the primary caregivers (93.8%) were female. All primary caregivers self-identified as a member of a racial/ethnic minoritized group. In addition, 54.7% were born outside the United States, 57.8% were married, and 56.4% had a college degree or higher. Income-to-needs ratio (INR) indicated that most of the families (62.3%) were classified per federal criteria as living in “extreme poverty” or were classified as “poor” or having “low income.” See Tables 1, 2 for additional demographic characteristics.

**Table 1:** iPCIT treatment group sociodemographic characteristics.

Sociodemographic characteristics	Full sample (*N* = 65)	
	*N*	%
Caregiver gender		
Female	61	93.8
Male	4	6.2
Caregiver ethnicity		
Hispanic	48	73.8
Non-Hispanic	17	26.2
Caregiver race		
White	52	80.0
Black/African American	11	16.9
Asian	1	1.5
Other	1	1.5
Caregiver nativity^a^		
U.S. born	29	45.3
Foreign-born	35	54.7
Caregiver region of origin		
North America	30	46.2
South American	17	26.1
Caribbean	12	18.5
Central America	5	7.7
Europe	1	1.5
Asia	0	0.0
Caregiver education^a^		
Junior high school (grades 6–8)	1	1.6
Some high school (grades 9–12)	4	6.3
High school degree/GED	10	15.6
Some college/Technical school	13	20.3
College graduate degree	20	31.3
Some post-graduate/professional	4	6.3
Graduate/professional degree (master's or beyond)	12	18.8
Caregiver current work status^a^		
Full-time employment	21	32.3
Part-time employment	18	27.7
Student	4	6.2
Homemaker	15	23.1
Unemployed	6	9.2
Income to needs ratio^b^		
Extreme poverty	3	4.9
Poor	15	24.6
Low income	20	32.8
Adequate income	12	19.7
Affluent	11	18.0

^a^ Based on *n* = 64 primary caregivers who reported data.

^b^ Based on *n* = 61 primary caregivers who reported data.

**Table 2:** iPCIT treatment group baseline measure characteristics.

Measure	*N*	Mean	SD
BDI developmental quotient	60	75.58	9.99
CBCL externalizing *T* score	65	67.43	6.65

BDI, battelle developmental inventory; CBCL, child behavior checklist.

### 2.2 Study design

All procedures were approved by the Florida International University Institutional Review Board. Each participant provided their written informed consent to participate in this study. Subsequently, initial baseline evaluations were conducted and then families were randomly assigned by a masked statistician to up to 20 weeks of iPCIT or RAU. As part of study participation, families randomized to iPCIT were provided with an earpiece and a tablet to facilitate home-based videoconferencing for their telehealth sessions. If families did not have access to a stable wireless network, a data plan was also provided. After completing major assessments (i.e., caregiver reports and observational tasks of family interactions) families received $100 via a gift card. Caregivers completed baseline questionnaires online via the Research Electronic Data Capture Platform, while observational tasks of parent-child interactions were conducted in the family's home at baseline and week 20 (posttreatment). All participants completed treatment before the COVID-19 pandemic. The study flow and retention rates were consistent with other studies conducting home-based assessments [see (66)].

### 2.3 Treatment

Analogous to clinic-based PCIT, iPCIT (66) is composed of two phases: Child-Directed Interaction (CDI) and Parent-Directed Interaction (PDI), each with one teach session and several coach sessions. In the CDI phase, therapists teach caregivers to use skills during child-directed play that promote a warm, secure, and responsive caregiver-child relationship (e.g., labeled praises) to improve child behavior. Across the CDI phase, changes in the caregiver-child relationship are thought to occur via two components: (1) live coaching of caregivers using the skills in play during sessions, and (2) home-based practice of skills (i.e., homework) via “special time.” The practice of skills outside session provides an opportunity for caregivers to incorporate these skills into their daily life.

During the CDI teach session, caregivers learn the skills that will be used during coach sessions. Specifically, caregivers are taught to limit their use of “don’t” skills (i.e., questions, commands, and criticisms), while capitalizing on their use of “do” or PRIDE skills (Praising child behavior, Reflecting child statements, Imitating child play, Describing child actions, and showing Enjoyment) to increase positivity and warmth in play with the child. At the beginning of each coach session, therapists code caregivers during a 5 min period with their child to assess caregiver progress in skill use over time. Therapists then spend the rest of each CDI session coaching caregivers on the use of these skills and assign homework to practice the skills daily at home during five minutes of special time with their child. During the PDI teach session, caregivers learn to use effective discipline strategies, such as implementation of effective commands and consistent follow-through with timeout for noncompliance, which are practiced during coach sessions. In PDI, caregivers are instructed to engage in homework in between sessions by continuing to implement special time with their child daily, as well as practicing the use of commands and timeouts to increase overall child compliance.

Participating families received up to 20 weeks of iPCIT in English (*n* = 35) or Spanish (*n* = 30) depending on caregiver reported language of preference and were evaluated at pre and post treatment. During the first phase, CDI, the number of sessions was capped at 6 sessions (including CDI Teach). Families then moved on to the second phase, PDI, whether or not they met mastery/competency criteria. This approach allowed flexibility in number of sessions while ensuring families participated in the PDI phase in the larger clinical trial.

Caregivers connected via an encrypted web call with their therapist for 1-to-1.5-hour sessions every week. Therapists used a secure videoconferencing platform to remotely conduct coaching of caregiver-child interactions in real time. Therapists were psychology doctoral students or post-doctoral fellows who completed PCIT training and received ongoing supervision and consultation from a PCIT International Global Trainer and the last author.

### 2.4 Measures

#### 2.4.1 Homework engagement

During weekly coach sessions for both CDI and PDI, caregivers reported on how many days they were able to practice special time since their last session. Families received up to 20 weeks of treatment, which was not based on graduation criteria. Thus, homework engagement was operationalized as a proportion score defined as the total number of days caregivers reported practicing special time divided by the total number of days available to practice in between sessions. Using a proportion to measure homework completion accounted for variability in number of treatment sessions across families and provided a standardized metric for engagement. Accordingly, these scores were not impacted by differing lengths of treatment and could be directly compared across participants. Homework engagement scores could range from 0 to 100%, with higher scores indicating higher levels of homework engagement. Additionally, we descriptively reported some of the self-reported barriers that impeded caregivers from completing homework. Caregivers selected whether one or several of these barriers affected their weekly engagement in treatment: “Nothing got in the way of completing homework,” “I felt too busy,” “I was out of town and physically away from my child”, “I forgot,” “My child was sick,” or “Other” with a box to explain the reason in writing.

#### 2.4.2 Acculturation and enculturation

The Abbreviated Multidimensional Acculturation Scale (AMAS) is a 43-item self-report measure that operationalizes acculturation and enculturation and was administered at baseline. The AMAS conceptualizes acculturation and enculturation separately across three dimensions: cultural identity, cultural knowledge, and language knowledge [for a total of 6 subscales (80)]. Examples of items in the acculturation subscale across each of the three dimensions include the following: “I have many close friends who are U.S. American.” “I enjoy watching U.S. American TV programs,” and “I am comfortable speaking English in social settings.” Examples of items in the enculturation subscale include: “I feel proud to be a part of my heritage culture,” “I often participate in traditions or celebrations from my culture of origin,” and “I enjoy listening to music in my heritage language.” Questions use a Likert-scale from 1 (Strongly disagree) to 4 (Strongly agree). Total acculturation scores, combining scores from all dimensions reflecting the individual's adaptation to the mainstream culture, and total enculturation scores, combining all scores from dimensions reflecting the individual's retention of their heritage culture, were used in analyses. Higher scores on these scales indicates stronger adaptation to mainstream culture and stronger retention of and engagement with their native culture, respectively. This questionnaire has shown to exhibit strong construct validity and reliability in assessments of internal consistency (Cronbach's alpha coefficients typically exceeding .80) and test-retest reliability across different ethnic groups in community and clinical samples (80, 81). Internal consistency in the current study was very good (*α*_Acculturation_ = .89, *α*_Enculturation_ = .86).

### 2.5 Statistical analysis

To evaluate the effect of acculturation and enculturation on caregivers' engagement with homework assignments during treatment, we conducted linear regression models using the overall average homework engagement proportion across treatment. The decision to use the overall average homework engagement measure for the primary analysis was driven by several factors: the variability in session attendance across participants, the relatively fewer opportunities to complete homework during the CDI phase, and the focus on sustained caregiver engagement throughout treatment.

Given that individuals simultaneously exhibit high levels of both acculturation and enculturation, low levels of both, or high levels of one and low levels of the other, we aimed to capture the complex and dynamic nature of the acculturation and enculturation processes through conducting three separate linear regression analyses using IBM SPSS Statistics (version 28.0). As shown in Table 3, Models 1 and 2 independently examined the effects of acculturation and enculturation on homework completion. Additionally, to account for the bidimensional nature of these constructs (e.g., caregivers can experience high levels of both acculturation and enculturation simultaneously), and to examine the unique contribution of one predictor over and above the other, we included both acculturation and enculturation as predictors in Model 3.

**Table 3:** Acculturation and enculturation as predictors of CDI homework engagement across treatment in caregivers of children with DD.

Predictor	Unstandardized coefficients		Standardized coefficients			95% CI	
	B	Std. Error	Beta	*t*	*p*-value	Lower bound	Upper bound
Model 1: Acculturation as a predictor							
Caregiver nativity	−.004	.075	−.008	−.049	.961	−.155	.147
Caregiver language of preference	.057	.083	.119	.689	.493	−.109	.223
Adequate/affluent income	.052	.066	.107	.792	.432	−.080	.185
Non-employed work status	.073	.063	.150	1.168	.248	−.053	.199
**Acculturation**	.110	.056	.342	1.971	.054	−.002	.222
Model 2: Enculturation as a predictor							
Caregiver nativity	.073	.069	.154	1.054	.297	−.066	.212
Caregiver language of preference	−.055	.073	−.115	−.748	.458	−.202	.092
Adequate/affluent Income	.085	.064	.175	1.336	.187	−.043	.213
Non-employed work status	.126	.064	.258	1.961	.055	−.003	.254
**Enculturation**	.140	.050	.394	2.824	.007*	.041	.239
Model 3: Acculturation and Enculturation as predictors							
Caregiver nativity	.037	.074	.078	.499	.620	−.112	.187
Caregiver language of preference	−.003	.084	−.006	−.034	.973	−.170	.165
Adequate/affluent Income	.074	.064	.151	1.148	.256	−.005	.202
Non-employed work status	.123	.064	.251	1.920	.060	−.005	.251
**Acculturation**	.072	.056	.223	1.279	.206	−.041	.184
**Enculturation**	.121	.051	.340	2.344	.023*	.017	.224

Excluded reference groups: Full-Time Employment (Work Status) and Low Income (INR), serving as baselines for comparison.

* *p* < .05.

Prior to conducting the regression analyses, we verified that the assumptions of linear regressions were met. We examined for linearity, independence of error, homoscedasticity, outliers, and normality of residuals. Scatterplots examining the relationships between homework engagement rates and both acculturation and enculturation scores were approximately linear. Furthermore, the Durbin-Watson statistic to assess for independence of errors in the regression models indicated independent residuals given values of 2.054 for acculturation and 1.845 for enculturation. Additionally, scatterplots and histograms showed a random distribution, indicating homoscedasticity. Normal probability plots of the residuals approximated a normal distribution, supporting this assumption. No outliers were found in the data. Lastly, Variance Inflation Factor (VIF) values were examined to check for multicollinearity among the predictors and covariates. All VIF values were below 10, indicating no issues with multicollinearity.

#### 2.5.1 Covariates

Nativity, caregiver language of preference, income-to-needs ratio (INR) and current caregiver work status were used as covariates in the regression analyses to ensure that findings were not due to linguistic, economic, or employment-related factors and consistent with previous research (12). Nativity was assessed by determining whether the caregiver was born in the United States or outside of the United States. Nearly half (45.3%) of caregivers self-reported being U.S. born, while just over half (54.7%) reported being born in another country. To control for potential confounding effects of nativity on outcomes, it was included as a covariate given that exposure to cultural norms, length of time in the host culture, and context of upbringing may intersect with acculturation and enculturation (82).

Caregiver language of preference refers to the language in which caregivers felt most comfortable speaking, and thus the language in which sessions were conducted. A slight majority of caregivers (53.8%) reported English as their language of preference, while 46.2% reported Spanish.

Income-to-needs ratio (INR) is a measure of economic well-being relative to family size and composition and was calculated by dividing total household income by the Federal Poverty Threshold (FPT) for a given year and family size (83, 84). INR can be categorized as “extreme poverty” when INR ≤ 0.5, “poor” when 0.5 < INR ≤ 1, “low income” when 1 < INR ≤ 2, “adequate income” when 2 < INR ≤ 4, and “affluent” when INR > 4. Most families in this study (62.3%) were living in extreme poverty, poor, or low-income categories. INR was dummy-coded for inclusion in the regression analyses, to account for its categorical nature. Additionally, because of the uneven distribution of participants in each category, income categories that share similar socioeconomic implications were grouped to avoid instability in regression estimates, inflated standard errors, and to improve statistical power. Extreme poverty (*n* = 3), poor (*n* = 15), and low income (*n* = 20) were grouped into a single “poor/low income” variable, reflecting individuals facing significant socioeconomic challenges or struggles with meeting basic needs. Adequate (*n* = 12) and Affluent (*n* = 11) were grouped into a single “adequate/affluent income” variable, reflecting individuals with fewer socioeconomic constraints.

Caregiver work status was also assessed using a categorical variable with five groups: full -time employment (*n* = 21), part-time employment (*n* = 18), student (*n* = 4), homemaker (*n* = 16), and unemployed (*n* = 6). Given the variability of number of participants in each category, work status was also grouped into two dummy-coded variables. Full-time and part-time were grouped into a single “employed” variable, while student, homemaker, and unemployed were grouped into a single “non-employed” variable.

## 3 Results

### 3.1 Descriptive statistics

Descriptive statistics including the mean, standard deviation, and range were computed for acculturation, enculturation, and homework engagement (see Table 4). The minimum rate of homework engagement was 0%, and the maximum rate was 95%. The average percentage of homework engagement across treatment was 51.87% (SD = .24), meaning that families, on average, completed special time at home on roughly half of the days that they were in treatment.

**Table 4:** iPCIT treatment group descriptive statistics.

Measures	*N*	Minimum	Maximum	Mean (SD)
Homework engagement^a^	65	.00	.95	.52 (.24)
Acculturation	64	1.28	4.00	3.00 (.76)
Enculturation	64	1.43	4.00	3.25 (.66)

^a^ Homework engagement reflects a percentage of completion from 0% (no completion) to 100% (full completion) throughout treatment.

In addition to asking caregivers to report their frequency of homework practice during the week, we asked caregivers to report any barriers or challenges to homework engagement. On average, most caregivers (54.28%) reported that “nothing got in the way of completing homework.” However, 16.20% of caregivers reported “feeling too busy.” Other reported challenges included “being out of town and physically away from their child” (4.28%), “forgetting to practice” (6.20%), and their “child being sick” (3.33%). Additionally, 15.71% of caregivers selected more than one of these reasons.

For acculturation and enculturation, the mean for Total U.S. Acculturation was 3.00 (SD = .76) and for Total Enculturation was 3.25 (SD = .66) with possible scores ranging from 1 to 4. The minimum score reported by caregivers on the total U.S. Acculturation subscale was 1.28, and on the total Enculturation subscale was 1.43.

### 3.2 Main findings

In the linear regression analyses, higher levels of acculturation did not significantly predict greater homework engagement (*B* = .110, *p* = .054), with a standardized *β* = .342, controlling for caregiver nativity, language of preference, INR, and current caregiver work status. The effect size, indicated by Cohen's *f*^2^, was 0.029, suggesting a small effect size. Higher levels of total enculturation significantly predicted greater homework engagement (*B* = .102, *p* = .007) with a standardized *β* = .394, controlling for caregiver nativity, language of preference, INR, and current caregiver work status. The effect size, indicated by Cohen's *f*^2^, was 0.104, suggesting a small to medium-sized effect of enculturation on homework engagement.

When acculturation and enculturation were included in the same model, acculturation did not significantly predict homework engagement (*B* = .072, *p* = .206), with a standardized beta of *β* = 0.223, controlling for caregiver nativity, language of preference, INR, and current caregiver work status. The effect size, indicated by Cohen's *f*^2^, 0.03 was small. Enculturation, on the other hand, significantly predicted greater homework engagement (*B* = .121, *p* = .023), with a standardized beta of *β* = .340, controlling for the same covariates. The effect size, indicated by Cohen's *f*^2^, was 0.10, suggesting a small to medium-sized effect.

## 4 Discussion

To our knowledge, the present study offers the first examination of the roles of acculturation and enculturation in predicting homework engagement among caregivers of children with DD participating in a telehealth behavioral parenting intervention. Given the many challenges faced by families from underserved racial/ethnic minoritized backgrounds in engaging with behavioral parenting interventions, this research sought to investigate how cultural factors might influence out-of-session engagement with treatment (i.e., homework participation), which in turn can promote more favorable treatment outcomes.

Throughout the entire treatment period, homework engagement averaged 51.87% (SD = .24). This level of engagement was comparable to what has been previously found in the literature (24–26), indicating that caregivers engaged in special time outside of sessions roughly half of the days in between treatment sessions. Additionally, in separate linear regressions, where acculturation and enculturation were independently explored, acculturation was not a significant predictor of homework engagement. However, enculturation was a significant predictor of homework engagement during iPCIT for this population. The small effect size for acculturation, and small-to-medium for enculturation, suggests that while both of these cultural processes show a positive trend towards homework engagement, other factors also play meaningful roles in determining out-of-session engagement.

When including both predictors together in the same linear regression model, acculturation was not a significant predictor, but enculturation significantly predicted homework engagement over and above the effect of acculturation. The significant effect of enculturation suggests that caregivers who identify more with their heritage culture are more likely to complete a greater percentage of homework across treatment. It is possible that cultural values and practices tied to enculturation, such as emphasis on collective well-being, respect, and family stability, could foster greater commitment to completing homework tasks to fulfill their role as responsible parents by prioritizing their child's needs and to ensure family well-being.

These findings underscore the importance of cultural factors in influencing treatment engagement, suggesting that both identification with the dominant culture and retention of one's cultural identity may be particularly important to consider for enhancing out-of-session engagement in remote iPCIT for caregivers of children with DD.

### 4.1 Acculturation and homework engagement

Inconsistent with our hypothesis, our results suggest that higher levels of *acculturation* are not significantly associated with greater homework engagement, suggesting that acculturation may not play as central a role in caregivers' ability to navigate the intervention process as initially expected. This finding contrasts with previous research suggesting that cultural adaptation and familiarity with mainstream therapeutic practices may facilitate treatment engagement and contribute meaningfully to better engagement and outcomes in behavioral interventions (10, 11). For example, McCabe and colleagues (10) presented a personalized parenting intervention approach, called PersIn, which utilized a pre-treatment culturally informed assessment to optimize cultural responsivity and tailor PCIT for each family. Although their approach assessed parenting values to inform cultural modifications, it notably did not include measures on acculturation. The present study's findings underscore the need to explore other cultural or systemic factors that may more directly affect caregivers' engagement in treatment, alongside further investigation into how acculturation might indirectly influence treatment processes.

### 4.2 Enculturation and homework engagement

Consistent with our hypotheses, we found that higher levels of *enculturation* were associated with increased homework engagement. Caregivers who reported maintaining a stronger connection with their cultural heritage demonstrated an increase in commitment to practicing iPCIT skills at home. This finding suggests that maintaining one's cultural identity may play an important protective role in supporting homework engagement in the context of telehealth parenting intervention for children with DD. While the effect size was small to medium, it indicates that enculturation might act as a protective factor, offering some level of support for caregiver homework engagement. This protective factor may potentially provide a framework that influences caregivers' motivation to engage in intervention tasks such as homework practice.

### 4.3 Acculturation, enculturation, and homework engagement

Although we predicted that having higher levels of acculturation and enculturation would predict higher rates of homework across treatment, the results suggested that when examined together, acculturation (adapting to the dominant culture) may not be as influential as enculturation (staying connected to the heritage culture) when it comes to homework engagement. This is an important distinction, as it shifts the focus to the value of maintaining ties to one's culture rather than assimilating to the dominant culture in determining engagement with treatment. These findings point to the importance of cultural fit in behavioral interventions. For example, if a caregiver's cultural identity aligns with the values and practices promoted in the treatment, they may be more engaged, as seen with enculturation. This suggests that interventions that acknowledge and integrate heritage culture might improve treatment outcomes, like increased homework engagement. In addition, given that enculturation had a more significant impact on homework engagement than acculturation when examined together, interventions should consider and focus more on how caregivers' cultural backgrounds, values, and practices influence their engagement with treatment. Tailoring treatments to reflect and respect these values may improve treatment adherence and outcomes.

### 4.4 Utilizing measures and treatment protocols to tailor treatment

This study highlights the importance of assessing enculturation to guide treatment. In both research and clinical practice, we often ask caregivers to complete multiple measures. Consistent with recommendations in the literature [e.g., (80, 85–88)], information from cultural assessments such as the AMAS and the Cultural Formulation Interview (CFI) (89) could be utilized to examine how the integration of cultural assessments can support therapists in providing more culturally aligned treatment to support families' homework engagement. For example, Sanchez and colleagues (86), found that, among families receiving services in Spanish, assessing cultural factors at the beginning of PCIT was associated with increased homework completion. Families who identify with more individualistic vs. collectivist values may benefit differently from treatment as a function of how much value they find in the skills being taught. Thus, understanding each family's views on parenting, sources of support, and most salient cultural values may provide useful directions for supporting out-of-session treatment engagement.

In addition to using cultural assessments to guide treatment planning, it is important to consider adapting the treatment protocol to accommodate cultural differences in caregiving practices (90). In fact, previous research has highlighted that caregivers from non-dominant cultures spend more time in treatment attempting to achieve CDI skills criteria, a challenge that is not always accounted for by existing treatment models (91). This finding suggests that current protocols may not fully align with the caregiving practices of families from diverse cultural backgrounds. Adaptations may involve modifying the expectations for skill acquisition to ensure the skills taught are meaningful within the context of a family's cultural values.

### 4.5 Caregivers of children with DD

Parenting perspectives are highly culturally dependent. Thus, caregiver's explanations or views as to what causes, maintains, and helps their child's behavioral challenges are critical for informing treatment planning for behavioral parenting interventions. Such views can influence treatment involvement and engagement, as well as caregiver beliefs about what kind of treatment and treatment tasks are most relevant for their child's difficulties (10). In fact, it has been shown that parents reporting lower acculturation agree less with therapists than parents reporting greater acculturation about the cause of their child's problems (88). Hence, focusing on enculturation-helping families embrace their cultural values and identity- could provide a meaningful avenue for enhancing treatment engagement, as our results suggest that fostering a sense of connection to cultural identity may positively influence homework engagement in treatment. In addition, caregivers from minoritized backgrounds utilize mental health services less often than White, non-Latinx/Latine caregivers, and, due to disparities and experiences with discrimination, often report greater levels of institutional mistrust. Thus, it is particularly critical to examine these factors in populations with high service needs, such as parents of children with DD, whose children often experience prevalence rates of behavioral problems two to four times higher than typically developing children (2, 92). This is the first study to directly examine cultural predictors of homework engagement in the context of telehealth parenting services for children with DD. Continued research in this area is critical to support positive, long-term, and culturally responsive improvements in this clinical population.

### 4.6 Clinical implications

These findings offer several implications for enhancing the effectiveness of behavioral parenting interventions, particularly in culturally diverse populations. Tailoring interventions to be informed by enculturation processes may improve engagement across the course of care. Culturally informed strategies that respect and integrate caregivers' cultural values and perspectives into the delivery of intervention practices could enhance out-of-session engagement. Specifically, therapists might benefit from systematically incorporating cultural assessments [e.g., Cultural Formulation Interview; (76, 85, 89, 92, 93, 94)] that address both the adaptation to mainstream therapeutic approaches and the maintenance of cultural identities.

Culture encompasses values, traditions, language, behaviors, communication styles, religion, societal norms, parenting styles, and more. Although culture is a very intricate concept of systems that are intertwined and unique to a given population, there are many studies that have specifically looked at the differences among cultures with regards to values and parenting styles (94). This information could be translated into practice and utilized to tailor treatment, particularly for behavioral parenting interventions. The incorporation of culturally relevant examples and practices into treatment could foster greater engagement and adherence (86). For instance, utilizing language/lingo relevant to a family's culture of origin can help promote enculturation within families, and in turn positively affect homework engagement. Adding culturally significant examples of “do skills” and “don’t skills” in daily situations that may be an important part of a families' daily living may also support the family's understanding of treatment skills in ways that are more consistent with their own worldviews and mindset. Parents in treatment who are showing limited increases in the amount of praise they are giving their child, for example, may benefit from being presented with examples of opportunities to praise child behaviors that are more directly in accordance with their cultural values.

In addition to incorporating cultural considerations, it is important to address barriers to homework engagement, such as time constraints and stress, through supportive interventions and resources that could further improve out-of-session participation rates. However, these barriers should not be conflated with cultural processes, such as acculturation or enculturation. Barriers to engagement refer to more practical issues that stem from the challenges of daily life (e.g., scheduling conflicts, other caregiving responsibilities, financial or emotional burdens). However, these barriers may be more common among minoritized families, adding to their levels of acculturative and daily stress. Families can problem-solve these factors with the help of the therapist, but only if they are inquired about and identified. For instance, when assessing barriers to homework engagement and treatment completion, clinicians should systematically ask whether treatment components and homework assignments align well with their values and parenting styles, while encouraging open-ended responses that allow families to discuss their unique circumstances. Assessing these barriers can help address implementation challenges, such as generational or cultural differences in parenting beliefs, that may affect homework engagement.

### 4.7 Limitations and future directions

This study has several limitations that must be considered. The effect sizes of acculturation and enculturation on homework engagement were relatively small. Thus, future research should investigate the effect of acculturation and enculturation on homework engagement in larger samples. Although this study provided significant effects for enculturation, it would be important to see if this effect size would be strengthened with a larger sample size.

Due to limited statistical power, we collapsed across racial/ethnic groups, preventing subgroup comparisons. An important future direction would be to conduct analyses with larger samples, allowing for the separation of racial/ethnic groups (e.g., Latinx/Latine, African American/Black, Asian), to better capture the nuanced differences in how acculturation and enculturation processes manifest, such as racial trauma among Black families, for example. Other factors, such as the intensity of intervention, caregiver stress levels, and available social support, likely also contribute significantly to engagement outcomes given the small effect sizes. Exploring these additional factors, such as caregiver mental health, social support networks, attitudes towards treatment, caregiver language proficiency, perceived discrimination, involvement of other caregivers, and intervention fidelity could provide a more comprehensive understanding of the predictors of homework engagement. These factors could be separately examined as potential moderators, as they may interact with acculturation/enculturation to predict homework engagement.

The sample was limited to caregivers of children with DD who were participating in an internet-delivered format of PCIT. Therefore, the findings may not be generalizable to other formats of PCIT or to families with different characteristics. Additionally, while the study controlled for nativity, caregiver language of preference, income-to-needs ratio and caregiver work status, other socioeconomic factors and life stressors that might influence engagement were not fully explored. Another limitation to consider is that caregiver homework engagement was based on caregiver self-report, which may introduce bias. Future studies could explore more ecologically valid methods for measuring homework engagement to enhance accuracy and reduce reliance on self-reported data. For example, incorporating behavioral observations, such as asking the caregiver to record short videos or audio recordings of each of their homework practice sessions and upload them to a secure platform, could provide more objective assessments. Additionally, the use of ecological momentary assessments, where caregivers complete real-time surveys on their smartphones immediately after engaging in special time, could reduce recall bias.

In this study, we only focused on CDI homework engagement across the entire course of treatment, which includes both CDI homework during the CDI phase, and CDI homework during the PDI phase. Future research with a larger sample size could further investigate the use of separate proportion indexes to measure CDI homework engagement separately during the CDI and PDI phases, given the different lengths and goals of these treatment phases in iPCIT. Additionally, examining acculturation and enculturation as predictors of PDI-specific homework engagement (i.e., practicing commands and implementing time-out specific assignments) would be a valuable avenue to explore for future research. Although we utilized linear regressions for our analysis, future research with larger sample sizes could consider alternative statistical approaches, such as piecewise regression (with careful consideration of potential overfitting and generalizability issues), or structural equation modelling (SEM), which would allow for a more nuanced examination of phase-specific homework completion trajectories in PCIT, as well as providing an avenue to explore indirect effects and mediation.

Given the AMAS is composed of multiple subcategories, it would be interesting to examine these subcategories separately in relation to homework engagement, which may assist therapists in focusing on one cultural variable at a time to strengthen homework engagement. In addition, it may be important to examine different cultural measures besides the AMAS to see if the interaction between acculturation/enculturation and homework engagement still hold significance. Depending on the clinical population, these measures could include the Bidimensional Acculturation Scale (BAS) (95), Vancouver Index of Acculturation (VIA) (96), Bicultural Identity Integration Scale (BIIS) (97), and the Cultural Values Scale (CVS) (98). Examining measures that are specifically designed for particular populations, such as the Acculturation Rating Scale for Mexican Americans-II (ARSMA-II) (99) or the East Asian Acculturation Measure (EAAM) (100), also may provide insight into how to tailor homework engagement in iPCIT for specific cultural groups of caregivers.

### 4.8 Conclusion

This study contributes to our understanding of how cultural factors can influence homework engagement in telehealth parenting interventions for children with DD. These findings underscore the differential roles of cultural processes in treatment engagement, highlighting the importance of culturally informed practices in treatment planning. Given the critical role of homework in behavioral interventions, future research should continue to examine homework engagement as a distinct domain of treatment participation. This work will allow for a continued exploration of others key factors that may play important roles in promoting treatment participation and quality care. By recognizing and integrating caregivers' cultural contexts into intervention strategies, practitioners can better tailor care and support families in achieving personally relevant outcomes.

## Data Availability

The data analyzed in this study is subject to the following licenses/restrictions: Deidentified participant data can be requested by researchers whose proposed use of the data has been approved with a signed data access agreement. Requests to access these datasets should be directed to acafatti@fiu.edu.
